# Bloodstream infection burden among cancer clinic patients with PICC Lines: A prospective, observational study

**DOI:** 10.1017/ash.2023.289

**Published:** 2023-09-29

**Authors:** Jessica Bethlahmy, Hiroki Saito, Bardia Bahadori, Thomas Tjoa, Shereen Nourollahi, Mohamad Alsharif, Justin Chang, Linda Armendariz, Vincent Torres, Sandra Masson, Edward Nelson, Richard Van Etten, Syma Rashid, Raheeb Saavedra, Raveena D. Singh, Shruti Gohil

## Abstract

**Background:** Oncology patients are at high risk for bloodstream infection (BSI) due to immunosuppression and frequent use of central venous catheters. Surveillance in this population is largely relegated to inpatient settings and limited data are available describing community burden. We evaluated rates of BSI, clinic or emergency department (ED) visits, and hospitalizations in a large cohort of oncology outpatients with peripherally inserted central catheters (PICCs). **Methods:** In this prospective, observational study, we followed a convenience sample of adults (age>18) with PICCs at a large academic outpatient oncology clinic for 35 months between July 2015 and November 2018. We assessed demographics, malignancy type, PICC insertion and removal dates, history of prior PICC, and line duration. Outcomes included BSI events (defined as >1 positive blood cultures or >2 positive blood cultures if coagulase-negative *Staphylococcus*), ED visits (without hospitalization), and unplanned hospitalizations (excluding scheduled chemotherapy hospitalizations). We used χ^2^ analyses to compare the frequency of categorical outcomes, and we used unpaired *t* tests to assess differences in means of continuous variable in hematologic versus solid-tumor malignancy patients. We used generalized linear mixed-effects models to assess differences in BSI (clustered by patient) separately for gram-positive and gram-negative BSI outcomes. **Results:** Among 478 patients with 658 unique PICC lines and 64,190 line days, 271 patients (413 lines) had hematologic malignancy and 207 patients (232 lines) had solid-tumor malignancy. Cohort characteristics and outcomes stratified by malignancy type are shown in Table 1. Compared to those with hematologic malignancy, solid-tumor patients were older, had 47% fewer clinic visits, and had 32% lower frequency of prior PICC lines. Overall, there were 75 BSI events (12%; 1.2 per 1,000 catheter days). We detected no significant difference in BSI rates when comparing solid-tumor versus hematologic malignancies (*P* = 0.20); BSIs with gram-positive pathogen were 69% higher in patients with solid tumors. Gram-negative BSIs were 41% higher in patients with hematologic malignancy. Solid-tumor malignancy was associated with 4.5-fold higher odds of developing BSI with gram-positive pathogen (OR, 4.48; 95% CI, 1.60–12.60; *P* = .005) compared to those with hematologic malignancy, after adjusting for age, sex, history of prior PICC, and line duration. Differences in gram-negative BSI were not significant on multivariate analysis. **Conclusions:** The burden of all-cause BSIs in cancer clinic adults with PICC lines was 12% or 1.2 per 1,000 catheter days, as high as nationally reported inpatient BSI rates. Higher risk of gram-positive BSIs in solid-tumor patients suggests the need for targeted infection prevention activities in this population, such as improvements in central-line monitoring, outpatient care, and maintenance of lines and/or dressings, as well as chlorhexidine bathing to reduce skin bioburden.

**Figure:**
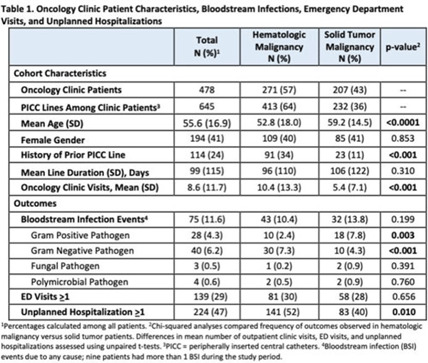

**Disclosures:** None

